# Forensic odontological observations in the victims of DANA air crash

**DOI:** 10.11604/pamj.2015.20.96.5360

**Published:** 2015-02-04

**Authors:** John Oladapo Obafunwa, Victor Olabode Ogunbanjo, Ogunbiyi Babatunde Ogunbanjo, Sunday Sokunle Soyemi, Francis Adedayo Faduyile

**Affiliations:** 1 Office of the Chief Medical Examiner, Department of Pathology and Forensic Medicine, Lagos State University College of Medicine and Teaching Hospital, Ikeja, Lagos, Nigeria; 2 Department of Oral Maxillofacial Surgery, Lagos State University College of Medicine and Teaching Hospital, Ikeja, Lagos; 3 Department of Child Dental Health, Lagos State University College of Medicine and Teaching Hospital, Ikeja, Lagos

**Keywords:** identification, forensic, dental records, fracture, mandible, maxillae

## Abstract

**Introduction:**

Forensic odontology or forensic dentistry is that aspect of forensic science that uses the application of dental science for the identification of unknown human remains and bite marks. Deaths resulting from mass disasters such as plane crash or fire incidence have always been given mass burial in Nigeria. This was obviously due to the fact that Forensic Pathologists whose roles involve disaster victim identification were not available at that time. However, in the DANA air crash in Lagos in 2012, the Forensic pathologist and dental teams were invited for the first time to identify the victims. The objectives of this paper are to identify the extent of victims’ identification using Forensic odontology alone and its combination with DNA analysis. It also presents the pattern of fractures seen in the mandible and maxilla of the victims.

**Methods:**

The bodies were dissected using following the standard protocol dissection. Prior to this all the victims had Dental Radiological Examination. The oral cavities were exposed after which the Odontology team was invited for photographing first, followed by dental charting. Fractures of the mandible, maxilla including the anatomical regions were all recorded and photographed. Dental prosthesis, restorations, crowns and bridge and other findings were also noted, recorded and compared with ante mortem records where available.

**Results:**

A total of152 bodies were recovered from the crash site while 148 victims were eventually identified through a combination of DNA analysis and forensic odontology. This represented 97.4%. Forensic odontology was the primary identifier in 10%. There were no fingerprinting information in this country at present therefore, it could not be used. A total of 89 (60%) were males while females accounted for 59(40%). This gives a ratio of 1.5:1. Most of the victims were in the age group 30-49years; this represented 52% of the victims while the least involved age groups were victims above 60 years of age which accounted for only 4.7%. Mandibular fractures were seen in 29 victims, maxilla in 15, combined mandibullo/maxillary in 15 victims, while 89victims had nojaw fracture. The most common area of fracture in the mandible was the body which accounted for 36.4%, closely followed byparasymphysealregion 31.9%, symphyseal 22.7% and the angle 9.0%. The most common fracture in the maxillae was palatal split fracture which accounted for 52%, this was followed by pterygoid 24%, alveolar 8% and multiple locations 16%.

**Conclusion:**

A combination of DNA analysis and forensic odontology was able to identify a total of 148 victims out of 152 representing 97.4%. Forensic odontology was the primary identifier in only 10%. In the latter, poor and lack of dental records were responsible for this very low figure. The most common area of fracture in the mandible was th ebody which accounted for 36.4%, while that of the maxillae was palatal fracture which accounted for 52%. Padding of the back of the seats in the aircraft should be canvassed for to provide Cushing effect for passengers.

## Introduction

Forensic odontology or forensic dentistry is that aspect of forensic science that uses the application of dental science for the identification of unknown human remains and bite marks. It plays an important role in the identification of victims of mass casualties such as airplane crashes, terrorist attacks, fires, floods, and earthquakes and other disasters [1]. The comparison of antemortem and post mortem dental records in criminal investigation, mass disasters and grossly decomposed bodies for positive identification has longed been proved. This type of identification is cheap, fast and cost effective [2]. Teeth and dental restorations are resistant to destruction by fire and are therefore useful in identification in cases of charred and severely decomposed bodies. Ante mortem and post mortem dental radiographs and charting are also compared. This again assists in the identification process [3, 4]. Different studies on the pattern of mandibular fractures in various countries have been previously reported. These are common injuries seen by oral andmaxillofacial surgeons with an incidence of 30% [5]. Anatomically, fractures of mandible are classified into regions namely; condyle, coronoid, ramus, angle, body, parasymphyseal, symphyseal and dentoalveolar regions [5]. The objectives of this paper are to identify the extent of victims’ identification using forensic odontology alone and/or its combination with DNA analysis. It also presents the pattern of fractures seen in the mandible and maxillae of the deceased.

## Methods

Consent was obtained from the department for the purpose of publication. The bodies were dissected using the standard dissection protocol. Prior to this all the victims had Dental Radiological Examination. The oral cavities were exposed after which the Odontology team was invited for photographing first, followed by dental charting. Fractures of the mandible, maxillae including the anatomical regions were all recorded and photographed. Dental prosthesis, restoration, crowns and other findings were also noted and recorded by the dental team. Thus, each victim had a full radiological examination, complete autopsy and dental works.

## Results

A total of 152 bodies were recovered from the crash site while 148 victims were eventually identified through a combination of DNA analysis and forensic odontology. This represented 97.4%. Forensic odontology was the primary identifier in 10%. There were no fingerprinting information in this country at present therefore, it could not be used. A total of 89 (60%) were males while females accounted for 59 (40%). This gives a ratio of 1.5:1. Most of the victims were in the age group 30-49 years; this represented 52% of the victims while the least involved age groups were victims above 60 years of age which accounted for only 4.7%. Fractures of the mandible were seen in 29 victims, maxillae in 15 and both fractures in 15 victims. Altogether 59 victims had one or more fractures while 89 victims had no fracture at all. This represented approximately 40% of the victims. The most common area of fracture in the mandible was the body which accounted for 36.4%, closely followed by parasymphyseal region 31.9%, symphyseal 22.7% and the angle 9.0%. The most common fracture in the maxillae was palatal split fracture which accounted for 52%, this was followed by pterygoid 24%, alveolar 8% and multiple locations16%.

## Discussion

This paper has attempted to identify the extent of victims’ identification using forensic odontology alone and its combination with DNA analysis. It has also presented the pattern of fractures seen in the mandible and maxilla of the victims. Although, there is variability in the distribution of the fractures of the mandible, the body was the most common fractured site in this study. This finding is seen to be at variance with earlier studies by many authors which reported the angle as the most frequently affected site [5–7]. Others have reported that the parasymphyseal part was the most common site [8–12].

This twist might be due to the fact that most of the earlier studies found in the literature dealt with road traffic accidents and fall from height, whereas this study mainly focused on victims of air crash. We are not unaware of the fact that the back of the seats in the planes are not usually padded. It will also be interesting to know the seating arrangements of the victims who had mandibular, maxillary and combined fractures. Future studies would attempt to relate victims assumed seat positions to the injuries sustained.

In a mass disaster involving a train accident in Zagreb, Croatia, only 5% of the victims were identified whereas 33% and 100% of British and Slovenian nationals were identified respectively in a plane collision by dental means alone [13]. In the former, there were no antemortem dental records of the victims while the latter had good dental records which yielded the high identification rate. Considering the increasing trend of spike in natural and man- made disasters, there is need for proper dental record keeping by general dental practitioners in both government and private facilities. A policy where all patients in government hospitals are made to have a panoramic dental radiograph will be a step in the right direction.

Similarly, in the Thai Tsunami of 2004, 61% of the victims were identified solely by dental methods, 1.3% by DNA,19% by fingerprinting information and 18% by the combination of more than one type [14]. This was also the pattern seen when the Danish team went into Thailand after the Tsunami to help with identification. The team worked from December 2004 to June 2005 and was able to identify 70.3% of Denmark nationals with the use of forensic odontology alone, and another 5.4% in combination with fingerprinting information. Fingerprinting was used in only 21.6% and DNA was used inonly one identification in combination with fingerprinting. Only one Danish individual was not identified [15] in that study. However, this study has revealed a great contrast in the identification where only 10% was identified using odontological method and another 97.4% using a combination of odontology with DNA method. The striking difference in the percentage of dental identification was due to the culture of not visiting the dentist in (this centre) this part of the world and partly due to poor record keeping of those who had visited the dentist. It is recommended that efforts should be made to encourage people to regularly visit the dentist so as to have dental records and radiograph which are to be kept by the dental surgeon in both government and private hospitals in addition to the establishment of adequate database. The large amount of resources spent on identification through DNA would have been better utilized on other valuable ventures. It is also further recommended that adequate padding of the back of the seats in the aircraft to provide Cushing effect for passengers should be canvassed for.

## Conclusion

A combination of DNA analysis and forensic odontology was able to identify a total of 148 victims out of 152 representing 97.4%. Forensic odontology was the primary identifier in only 10%. In the latter, poor and lack of dental records were responsible for this very low figure. The most common area of fracture in the mandible was the body which accounted for 36.4%, while that of the maxillae was palatal fracture which accounted for 52%. People should be encouraged to regularly visit the dentist so as to have dental records and radiograph which are to be kept by the dental surgeon in both government and private hospitals in addition to the establishment of adequate database. Padding of the back of the seats in the aircraft should be canvassed for to provide Cushing effect for passengers.
